# Antitumoral-Embedded Biopolymeric Spheres for Implantable Devices

**DOI:** 10.3390/pharmaceutics16060754

**Published:** 2024-06-03

**Authors:** Valentina Grumezescu, Oana Gherasim, Bianca Gălățeanu, Ariana Hudiță

**Affiliations:** 1 Lasers Department, National Institute for Lasers, Plasma and Radiation Physics, 077125 Magurele, Romania; 2 Department of Biochemistry and Molecular Biology, University of Bucharest, 050095 Bucharest, Romania

**Keywords:** biodegradable coatings, methotrexate, PLGA spheres

## Abstract

The bioactive surface modification of implantable devices paves the way towards the personalized healthcare practice by providing a versatile and tunable approach that increase the patient outcome, facilitate the medical procedure, and reduce the indirect or secondary effects. The purpose of our study was to assess the performance of composite coatings based on biopolymeric spheres of poly(lactide-co-glycolide) embedded with hydroxyapatite (HA) and methotrexate (MTX). Bio-simulated tests performed for up to one week evidenced the gradual release of the antitumor drug and the biomineralization potential of PLGA/HA-MTX sphere coatings. The composite materials proved superior biocompatibility and promoted enhanced cell adhesion and proliferation with respect to human preosteoblast and osteosarcoma cell lines when compared to pristine titanium.

## 1. Introduction

Primary (bone-originating tumors) and secondary (metastatic lesions) bone cancers pose a major treat in the worldwide healthcare system. Primary malignant bone tumors, or bone sarcomas, are rare and aggressive conditions that represents less than 1% of all cancers and up to 5% of childhood malignancies, mainly affecting young and adult active individuals with a 5-year survival rate of ~70% [1,2]. Secondary malignant bone tumors accompany other organ malignancies (mostly lung, breast, prostate, and kidney carcinomas), prevailing in adult and elderly population and associated with high morbidity and bad prognosis [3,4].

Though important progress has been clinically reported in the management of bone cancer, its interdisciplinary challenge still requires surgery along with the insertion of implantable devices and in conjunction with combined therapy (chemotherapy and radiotherapy). Owing to their intrinsic biocompatibility, superior biomechanics, and long-term general stability, titanium-based biomaterials are indisputable candidates for the fabrication of bone fixation devices [5,6], bone implants and prostheses [7,8]. Moreover, the compositional and microstructural versatility of such biomaterials, together with their native biochemical reactivity, facilitate the successful development of advanced bone adjuncts and substitutes [9,10].

Bone reconstruction and augmentation following cancer-related resection is a current challenge of modern healthcare practice, as biomechanical failure, opportunistic infection, and tumor recurrence can alter the healing process [11,12]. Besides conventional cementation, the biomimetic modification and coating of metallic surfaces provide an attractive strategy to modulate their bioactivity, considering that an accelerated and complication-free osseointegration is essential for the stabilization and long-term performance of bone implantable devices [13,14]. Although comprehensive studies explore the prospective local management of periprosthetic infection [15,16] and tumor recurrence [17,18] through multifunctional interfaces, high-dosage and multiple systemic treatments are employed to fight against these complications in a partially selective manner and with reduced therapeutic efficacy. 

The surface coating of titanium-based devices by calcium phosphates provides a suitable approach to overcome their limited bioinertness and modulate their osseointegration. Given its compositional and microstructural similarity with the bone apatite, synthetic hydroxyapatite (HA) is an extensively explored calcium phosphate representative for bone-related applications [19,20]. The HA coating of metallic surfaces has been validated as an efficient strategy to improve post-implantation osseointegration, stabilize bone growth around the implanted device, and accelerate bone regeneration [21,22].

The nanosize-governed reactivity and bioactivity of HA is beneficial for protein adsorption and cell adhesion, further leading to increased proliferation and decreased apoptosis in osteoblasts, up-regulated differentiation in osteoprogenitor cells, and down-regulated events in osteoclasts [23,24,25]. In addition to its excellent biocompatibility and tunable biodegradability, the unique composition-guided and structure-related versatility of HA paves the way towards the design and development of personalized platforms for bone cancer management. 

The osseointegration of titanium-based biomaterials can be substantially improved through composite or hybrid polyester/HA coatings, as these highly biocompatible and application-related biodegradable and bioresorbable polymers have beneficial effects on the repair and regeneration of bone tissue [26,27]. Along with these characteristics, the tunable solubility, degradation, thermoplasticity, and hydrophilicity/hydrophobicity of poly(lactide-co-glycolide), PLGA, facilitate the fabrication of modern biomedical platforms including stimuli-responsive pharmaceutical formulations, personalized implantable devices, and tissue substitutes [28,29,30]. Given their impressive ability to improve the osseointegration process [31,32] and to enable the local release of bioactive substances in a controlled and targeted manner [33,34], PLGA/HA composites are potent candidates for the local management of bone tumors [35,36]. 

To prevent bone tumor recurrence, perioperative chemotherapy and/or radiotherapy is currently recommended following the implantation of metallic devices. To overcome the limitations of conventional chemotherapy—such as reduced local drug availability, low patient compliance, and systemic side effects [37,38]—and concomitantly provide an enhanced osseointegration, the surface modification of metallic implantable devices with osteoconductive or osteoinductive coatings able to exert local chemotherapy is a current challenge in modern medicine. 

Therefore, this study reports the development of coatings based on PLGA/HA spheres loaded with methotrexate (MTX), a potent folate derivative with antineoplastic and immunosuppressant effects [39]. Specific bio-simulated studies and complementary cellular assays have been considered to evaluate the ability of PLGA/HA-MTX coatings to boost the osseointegration of titanium-based implantable devices while providing local chemotherapeutic effects.

## 2. Materials and Methods

### 2.1. Materials

All chemicals required for the synthesis of nanostructured composites were purchased from Sigma-Aldrich/Merck (Darmstadt, Germany), namely poly(DL-lactide-co-glycolide) (PLGA) with lactide:glycolide of 65:35 (40,000–75,000 g/mol), methotrexate (MTX, 454.44 g/mol), polyvinyl alcohol (PVA, 87–89% hydrolysis degree), chloroform, Na_2_HPO_4_·2H_2_O, NaOH (10%), and ethanol (C_2_H_6_O). The same supplier provided all reagents required for obtaining the simulated body fluid (SBF), such as NaCl, NaHCO_3_, KCl, K_2_HPO_4_·3H_2_O, MgCl_2_·6H_2_O, HCl, CaCl_2_, Na_2_SO_4_, and (CH_2_OH)_3_CNH_2_.

For the biological investigations, the cell lines were purchased from American Type Culture Collection (ATCC, Manassas, VA, USA), while most of the reagents and cellular kits were purchased from Sigma-Aldrich (Steinheim, Germany), except for the Live/Dead kit and fetal bovine serum (FBS) that were acquired from ThermoFisher Scientific (Waltham, MA, USA).

### 2.2. Chemical Synthesis of PLGA/HA-MTX Coatings

The hydroxyapatite (HA) nanoparticles were prepared by the co-precipitation method, according to our previously published papers [40]. Briefly, a P-containing and a Ca-containing solution were mixed under magnetic stirring. After alkaline pH adjustment and one-day maturation, the final solution was filtered, triply washed, and dried.

The composite spheres based on PLGA biopolymer, HA nanoparticles, and MTX were prepared using the solvent evaporation method [41]. Briefly, a mixture consisting of PLGA, HA, and MTX in a 10:1:0.2 mass ratio was dispersed in chloroform by sonication. The PLGA/HA-MTX phase was emulsified for 8 min with a SONIC-1200WT sonicator purchased from MRC Scientific Instruments (Harlow, Essex, UK). The sonication was performed in ON/OFF steps of 7 s and 3 s, respectively, in a 5 mL aqueous phase containing 2% (*w*/*v*) PVA, with a maximum temperature limitation of 37 °C. The resulting emulsion was added in deionized water and stirred for 4 h until the chloroform was completely evaporated; then, it was centrifuged at 6000 rpm for 15 min. The obtained composites were washed three times in deionized water, collected by filtration, and then subjected to freeze-drying. 

The as-synthesized PLGA/HA-MTX materials were further used to prepare composite coatings by the dip-coating technique [42]. Medical-grade titanium discs (12 mm diameter, 0.1 mm thickness) were thoroughly cleaned and then immersed in PLGA/HA-MTX suspension. After 10 s, the discs were gradually withdrawn and placed in a laminar flow cabinet for 2 h, until the complete drying of the double-side coated titanium.

### 2.3. Physicochemical Investigation

To evaluate the release profile of MTX from the composite coatings, UV–Vis analysis was performed with Evolution 220 equipment (Thermo Scientific, Schwerte, Germany) operated with the Insight2 software.

Scanning electron microscopy (SEM) images were collected using the secondary electron beam (20 kV acceleration voltage) of an InspectS50 FEI equipment accessorized with an energy-dispersive X-ray spectroscopy (EDS) detector (Thermo Fisher Scientific, Hillsboro, OR, USA).

The compositional analysis was performed using an IRTracer-100 system (Shimadzu Europa GmbH, Duisburg, Germany). All scans were recorded in the 400–4000 cm^−1^ wavenumber range (4 cm^−1^ resolution) in the attenuated total reflection (ATR-FTIR) mode.

### 2.4. Testing the Drug Release and Coating Degradation

Before assessing the behavior of PLGA/HA-MTX coatings under biologically simulated conditions, the calibration curve of the antitumor drug was obtained. Solutions of MTX with 10, 12.5, 15, 20, 25, 30, 37.5, 40, and 50 μg/mL concentration were prepared in simulated body fluid (SBF, pH = 7.42) by magnetic stirring at room temperature, then analyzed in the absorbance mode with an Evolution 220 UV–Vis spectrophotometer. SBF was prepared after Kokubo’s recipe and was selected as the active testing medium thanks to the ion concentrations, which are similar to human blood plasma [43,44].

Additionally, the mass of PLGA/HA-MTX coatings (representing the average mass of 5 individual measurements per sample) was determined as the weight difference between coated and pristine (uncoated) titanium using the Mya 0,8/3.3Y precision balance (Radwag, Radom, Poland). 

For dynamic tests, samples with similar coating mass were placed in individual channels (containing 4 mL of SBF) of a multichannel cell (two samples per each testing point). Constant flow rate (1 mL/min) and testing temperature (37 °C) were achieved by coupling the multichannel cell to an Ismatec peristaltic pump (Cole-Parmer, Wertheim, Germany) and a Grant Instruments thermostatic water bath (Fisher Scientific, Vantaa, Finland), respectively. After dynamic testing for different time points (4, 8, 12, 24, 48, 72, 96, and 168 h), samples were removed, rinsed with deionized water, dried in a laminar flow cabinet, and weighed. 

The average mass variation (Δm) of PLGA/HA-MTX samples was estimated as the difference between initial and final coating masses, weighted before and after dynamic testing, respectively. To quantify the MTX levels released in SBF, the testing solutions from each channel were collected and subjected to UV–Vis analysis. Compositional and microstructural aspects on the dynamically tested PLGA/HA-MTX coatings were obtained by Fourier transform infrared spectroscopy (FTIR) and SEM studies, respectively. 

### 2.5. Biological Investigations

#### 2.5.1. Cell Culture Models

Human preosteoblast hFOB 1.19 (CRL-3602^TM^, ATCC) and human osteosarcoma Saos-2 (HTB-85^TM^, ATCC) cell lines were used for in vitro experiments. The hFOB 1.19 cell line was cultured in a 1:1 mixture of Ham’s F12 Medium and Dulbecco’s Modified Eagle’s Medium, with 2.5 mM L-glutamine, supplemented with 10% FBS, 1% antibiotic mixture, and 0.3 mg/mL G418. Cells were maintained at 34 °C in a humidified atmosphere with 5% CO_2_. The Saos-2 cell line was cultured in McCoy’s 5a Modified Medium, supplemented with 15% FBS and 1% antibiotic mixture, and maintained in standard cell culture conditions (37 °C, 5% CO_2_).

Both cell lines were routinely passaged using 0.25% trypsin-EDTA when reaching 80% confluence, a procedure that was also used for cell detachment before material seeding. Before cell seeding, materials were sterilized by UV exposure on both sides and aseptically transferred in 24-well culture cell plates. Uncoated titanium substrates were employed as experimental controls and were seeded and processed identically to PLGA/HA and PLGA/HA-MTX coatings. Cells were cultured at an initial density of 1 × 10^4^ cells/cm^2^ and left for 30 min to adhere to the substrate before being immersed in the appropriate complete growth medium and further incubated in appropriate cell culture conditions until sample processing for the following assays. 

#### 2.5.2. MTT Assay

To investigate the viability and proliferation of normal and tumor cells post contact with the PLGA/HA and PLGA/HA-MTX coatings, the colorimetric MTT assay was performed. Briefly, 24 h and 72 h after experiment initiation, the samples were transferred into a new cell culture plate and immersed in a freshly prepared MTT solution (1 mg/mL) for 4 h at 37 °C. After the incubation time expired, the MTT solution was discarded, and the resulting formazan crystals were solubilized in an appropriate volume of DMSO. The obtained solutions were measured at 550 nm using the FlexStation 3 Multi-Mode Microplate Reader (Molecular Devices, San Jose, CA, USA). The results are presented as % of cell viability considering the mean optical density (O.D.) obtained for the experimental control at 24 h as 100% of cell viability. 

#### 2.5.3. Live/Dead Assay

For a qualitative examination of the ratio between the live and dead cells following contact with the samples, as well as to visualize the cell distribution on the sample surfaces, Live/Dead staining was performed. In short, after 24 h and 72 h, the samples were washed with PBS and immersed in the Live/Dead staining solution that was freshly prepared following the manufacturer’s instructions. After a 20 min incubation in the dark at room temperature, the samples were investigated in fluorescence using the Olympus IX73 microscope and CellSense F software v8.0.2. which was used for image capture, processing, and analysis. 

#### 2.5.4. Cytoskeleton Visualization

To assess cell adhesion and morphology after 24 h and 72 h of cell contact with the PLGA/HA-MTX coatings, F-actin filaments were evidenced by fluorescence microscopy. Cell fixation and cell membrane permeabilization were conducted through consecutive incubations in a 4% paraformaldehyde (PFA) solution for 20 min and a 0.1% Triton X-100/2% bovine serum albumin (BSA) solution for 1 h. Fluorescent labeling of F-actin filaments involved staining with a fluorescein isothiocyanate (FITC)-conjugated phalloidin solution at 37 °C for 1 h, while a 4,6-diamidino-2-phenylindole (DAPI) solution was employed for nuclear staining. Fluorescence images were captured and analyzed with the Olympus IX73 microscope and CellSense F software v8.0.2. 

## 3. Results and Discussion

### 3.1. Bio-Simulated Behavior of PLGA/HA-MTX Sphere Coatings 

The successful use of PLGA in developing biodegradable and bioresorbable formulations relies on the excellent biocompatibility and attractive characteristics of this copolymer, which can be tuned depending on its composition and microstructure. The physiologically comparable mechanical properties and physiologically triggered thermoplasticity, as well as the chemical reactivity, solubility and degradation of PLGA-based biomaterials represent indisputable advantages in fabricating modern biomedical platforms, including stimuli-responsive pharmaceuticals, performance-enhanced implantable devices, and personalized tissue substitutes [45,46,47]. More than representing effective carriers for the antitumor cargo (limiting or preventing its degradation, potentiating its therapeutic effect, and providing local action due to enhanced permeability and retention effect) [48,49], the additional (bio)chemical tuning of PLGA particulate formulations (micro-/nano-sized particles, spheres, and capsules) enables the fabrication of advanced active platforms that specifically and selectively bind to cancerous cells (by targeting overexpressed surface molecules and/or cellular receptors) [50,51] and circumstantially exert their effects under combined therapy [52,53].

Developing MTX carriers for bone cancer is a current challenge of the healthcare practice, as the optimal formulation should preserve the high potency of this anti-metabolite drug against tumor cells while limiting collateral toxicity and immunosuppression, preventing the inhibition of normal nucleic acids synthesis, and avoiding drug resistance occurrence [54,55]. To extend its clinical use in bone cancer management, MTX-loaded composites based on calcium phosphates and biopolymers have been proposed for a cancer-free bone repair [56,57]. Herein, composite formulations of PLGA and HA nanoparticles have been proposed for the entrapment and release of methotrexate.

**Figure 1:**
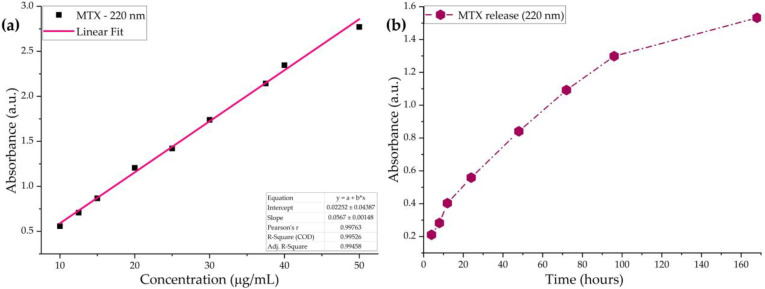
Graphic representation of the MTX’s calibration curve at 220 nm (**a**) and the release profile of MTX from PLGA/HA-MTX sphere coatings under bio-simulated conditions (**b**).

Before assessing the release kinetics of the antitumor drug from the PLGA/HA-MTX coatings under bio-simulated conditions, the graphic representation of concentration-depended absorbances of MTX solutions was used to plot the drug’s calibration curve (Figure 1a). Compliant with previous studies, the UV–Vis spectra of MTX solutions (prepared in fresh SBF) exhibited four specific bands between 200 and 400 nm [58,59] due to π → π* (220 and 258 nm) and n → π* (302 and 372 nm) transitions within conjugated carboxylates and amides. In our case, the calibration curve of MTX plotted for the 220 nm absorbance maxima in the 10–50 μg/mL concentration range showed good linearity, according to the Lambert–Beer law, with a correlation coefficient of 0.995.

After the dynamic testing of PLGA/HA-MTX-coated titanium, the release profile of MTX in SBF was determined by spectrophotometrically monitoring the variations in the 220 nm absorbance peak (Figure 1b), and the amount of released drug was estimated by considering the calibration curve of MTX. Throughout our experiments, a gradual release of the antitumor drug from the composite coatings was observed, with a continuous increase in the amount of released MTX ranging from ~3 μg/mL (4 h) to ~27 μg/mL (7 days). The concentration of MTX released in the first day of dynamic testing (24 h) was determined as 9.5 μg/mL, and more than half of the total released drug (14.4 μg/mL) occurred in the next day (48 h). Following additional testing, the concentrations of released MTX were estimated at 18.9 μg/mL (72 h) and 22.5 μg/mL (96 h). 

A sustained release of reduced MTX amounts was evidenced for up to 7 days, which is compliant with previous studies on the behavior of drug-loaded PLGA-based formulations under bio-simulated conditions [42,60]. A similar release profile of MTX was determined for PLGA-PEG (polyethylene glycol) and PLGA-PVA (using acid-terminated copolymer) nanocomposites at physiological pH values, with a cumulative release of 15% after 3 days [61] and 55% in 6 h [62], respectively. The prominent release of MTX was evidenced for PLGA/surfactant particles under neutral [63] and acidic [64] conditions, with an initial burst release in the first 12 h, followed by much higher levels of released MTX until day 2 and a gradual increase in the amount of released antitumor drug in the next days. Aiming for topical applications, moderate compositional and morphological alterations have been reported in PLGA (50:50) spheres and PLGA (65:35) coatings up to 21 and 15 days of bio-simulated evaluation, respectively, while the 65:35 (LA:GA) copolymer resulted in prominent copolymer degradation and drug release after 3 weeks [42,60]. Since the degradation and resorption of PLGA-based formulations should comply with the bone repair and regeneration kinetics (which includes long-term mechanisms), selecting a PLGA copolymer with extended degradation (and consequently prolonged drug release) is beneficial for bone tissue engineering applications [30,65]. Herein, selecting an LA-enriched PLGA representative determined a gradual and slow release of the antitumor drug due to the abundance of LA-originating hydrophobic methyl side groups and GA-mediated reduced crystallinity [66,67].

**Figure 2:**
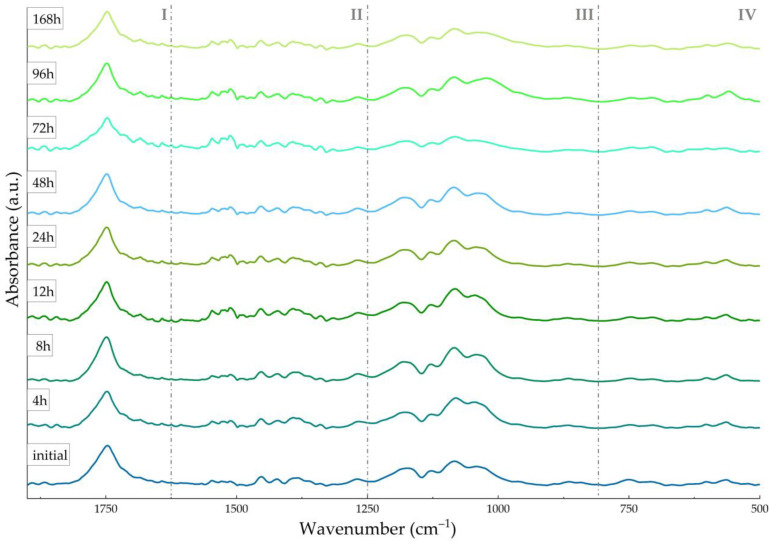
ATR-FTIR of PLGA/HA-MTX sphere coatings, before and after testing under bio-simulated conditions.

Comparative IR spectra of initial and dynamically tested samples were collected to investigate the composition of PLGA/HA-MTX coatings (Figure 2). 

The IR spectrum of the initial sample confirmed the formation of PLGA/HA-MTX composites by the clear presence of PLGA-originating (region I) and HA-originating (region III) moieties like symmetric C=O stretching (~1750 cm^−1^) [58,68] and asymmetric (~1130, ~1080, and ~1040 cm^−1^) and symmetric (~960 and ~860 cm^−1^) stretching of P–O [69,70], respectively. Other maxima, though reduced in intensity, were identified in the last spectral region, namely the copolymer’s C–H out-of-plane bending at ~750 cm^−1^ and the apatite’s O–P–O asymmetric deformation between 610 and 550 cm^−1^. Overlapped PLGA [71] and HA [72] vibrations were noticed in region III (C–O stretching at ~1180 cm^−1^) and region II (C–H asymmetric and symmetric deformation, and C–O bending at ~1450 cm^−1^), while region II also contained superposed MTX (carboxamide and aromatic C=C vibrations between 1550 and 1400 cm^−1^) [54,73] and PLGA (–CH_3_ and –CH_2_– bending at ~1380 cm^−1^) [74,75] vibrations. 

The IR spectra of PLGA/HA-MTX-coated titanium samples subjected to bio-simulated dynamic testing showed the presence of all previously identified moieties, indicating their preserved composition, with no drastic alterations or secondary products being observed after one week. With increasing the testing time, a reduction in the intensity of ester-originating carbonyl from PLGA (~1750 cm^−1^) was noticed, concomitant with the appearance of two distinctive peaks at ~1715 cm^−1^ and ~1680 cm^−1^, corresponding to conjugated carbonyls of carboxylic acids (LA and GA resulted from PLGA’s hydrolysis) [76] and MTX (due to the gradual exposure of the embedded antitumor drug) [77]. The sustained release of MTX following the SBF-mediated hydrolysis of PLGA was also supported by the more intense vibrations of carboxamide moieties (region II). Furthermore, reduced-in-intensity PO_4_^3−^ vibrations (regions III and IV) and slightly intensified carbonate vibrations (C–O stretching and bending in regions III and II) were observed, suggesting the formation of substituted apatite [78,79].

Our findings are in good agreement with those of other studies regarding the time-dependent bio-simulated behavior of composites based on PLGA and HA. For instance, the biomineralization and subsequent HA modification of PLGA and PLGA/silk porous microspheres have been evidenced after 24 h immersion in Ca^2+^-saturated solution and one week of incubation in SBF, respectively [80]. The superior mineralization capacity of PLGA microparticles [81], membranes [82], and scaffolds [83] under biologically simulated conditions has also been reported. The modification of the HA nanotube array with PLGA and sodium hyaluronate particles enabled the sequential release of immunomodulatory cytokines, being proposed as biomimetic bone-to-implant interfaces for the accelerated osseointegration of titanium implants [84]. The addition of nanosized calcium phosphates within PLGA [85], PLGA/polysaccharide [86], and PLGA/protein [87] scaffolds determined decreased porosity, increased mechanical properties, and reduced degradation rate when compared to bare biopolymer scaffolds, finally resulting in enhanced biomineralization and superior bone repair ability. 

**Figure 3:**
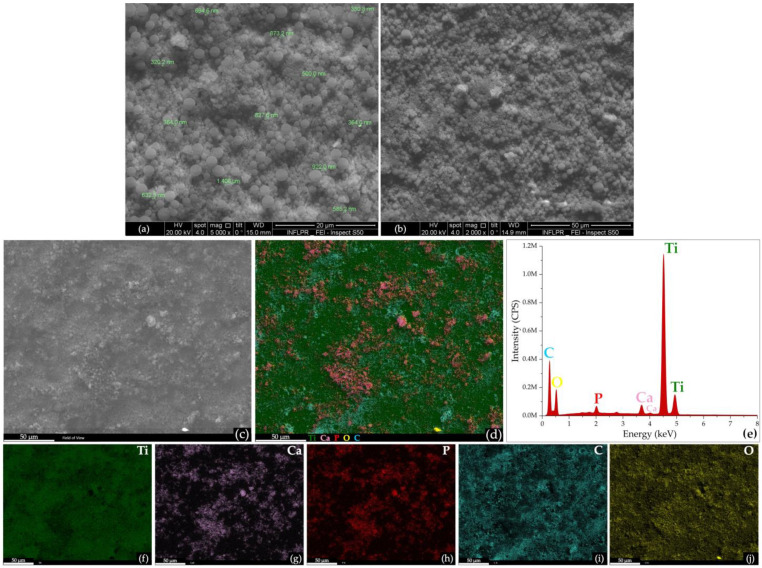
SEM micrographs of initial PLGA/HA-MTX sphere coatings (**a**,**b**), SEM image (**c**) and corresponding EDS spectrum (**e**), overlapped EDS map (**d**) and individual EDS maps (**f**–**j**) of initial PLGA/HA-MTX sphere coatings.

**Figure 4:**
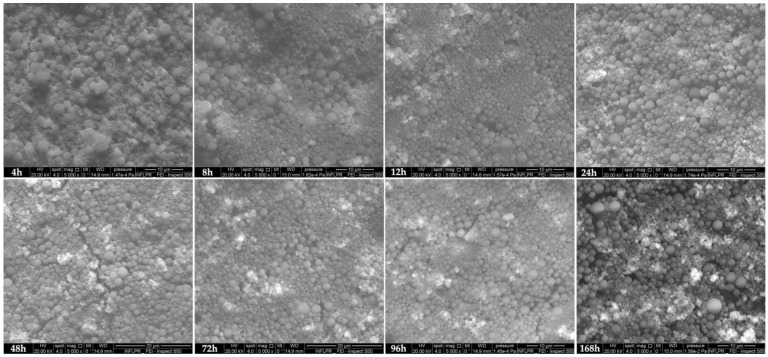
SEM micrographs of PLGA/HA-MTX sphere coatings after testing under bio-simulated conditions.

Taken together, the infrared data evidenced slight compositional modifications of the PLGA/HA-MTX coatings during dynamic testing under physiological conditions, confirming the gradual release of the antitumor drug and mostly evidencing reduced polymer degradation and the formation of secondary calcium phosphates. Further microstructural aspects on the PLGA/HA-MTX coatings, before (Figure 3) and after (Figure 4) bio-simulated evaluation, have been evidenced by SEM analysis.

Well-defined particles, with exclusive spherical morphology, smooth surface, and particle size between 300 nm and 2 μm, were obtained using the microemulsion protocol (Figure 3a). Compliant with the IR data, these results confirmed the successful incorporation of HA nanoparticles withing PLGA spheres, as no secondary structures were noticed at this level. Similar results have been reported for HA/PLGA (~250 nm) and PLGA/HA (3–8 μm) spheres synthesized by the modified Pickering emulsion [88], while PLGA/HA microparticles (~250 μm) have been successfully obtained by the airflow shearing method [89]. The PLGA/HA-MTX spheres were uniformly distributed on the surface of titanium substrate, adapting to the intrinsic topography of the metallic substrate, and resulting in continuous and homogenous coatings (Figure 3b), as evidenced at structural (Figure 3c) and compositional (Figure 3d) levels. Complementary EDS data (Figure 3e) showed the presence of characteristic HA elements, namely Ca (~3.7 and ~4 keV) and P (~2 keV) with a Ca/P atomic ratio of 1.54, while other identified elements were Ti (~4.5 and ~4.9 keV, substrate-originating), C (~0.2 keV, PLGA-originating), and O (~0.5 keV, originating from inorganic oxides, calcium phosphates, and copolymer). The elemental distribution maps confirmed the formation of composite spheres and revealed the uniform distribution of HA aggregates within the polymeric matrix (Figure 3f–j).

To evaluate the microstructural modifications of PLGA/HA-MTX sphere coatings during bio-simulated evaluation, SEM micrographs of initial samples (Figure 3) and samples subjected to physiologically simulated dynamic testing (Figure 4) were used. Compared to the initial coatings, similar microstructural aspects were noticed for PLGA/HA-MTX coatings after 4 h of dynamic testing. At 8 h and 12 h after immersion, a smoother surface of coatings was observed (most probably due to the moisture-mediated rearrangement of copolymer chains), concomitant with a slight and gradual shrinkage of the composite spheres. This behavior has been previously reported for bigger PLGA microparticles [90,91] and might have resulted from the release of surface-attached MTX and HA (thus supporting the presence of conjugated carbonyl and more intense amide vibrations and being compliant with the more intense phosphate vibrations recorded for these time points by ATR-FTIR, respectively). The local exposure of nano-HA occurred further, as evidenced by the abundance of bright regions recorded after 12 h and 24 h. After one day of bio-simulated evaluation, PLGA-based spheres seemed to recover their initial dimensions, following swelling. By increasing the testing time to 48 h, 72 h, and 96 h, microcracks and erosion pits were observed due to the physical and chemical degradation of coatings (time-depended mechanical damage and SBF-mediated hydrolysis and autohydrolysis of PLGA, respectively). These microstructural modifications contributed to the important MTX release that occurred from the second day of bio-simulated assessment. Also, more inorganic aggregates were identified at these testing time points, which might have simultaneously resulted from released HA and newly formed apatite (as their presence is predominantly noticed near the structural coating defects that are favorable for the local deposition of SBF-originating ions, followed by mineralization). More and larger coating defects were identified after one week, but also an increased dimension and distribution of inorganic aggregates were observed. 

**Figure 5:**
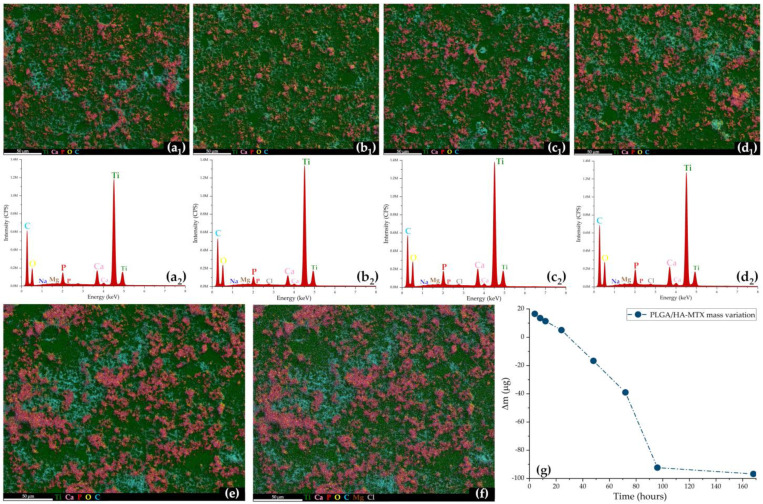
EDS maps (**a_1_**–**d_1_**,**e**,**f**) and EDS spectra (**a_2_**–**d_2_**) of PLGA/HA-MTX sphere coatings after testing under bio-simulated conditions at 24 h (**a_1_**,**a_2_**), 48 h (**b_1_**,**b_2_**), 72 h (**c_1_**,**c_2_**), 96 h (**d_1_**,**d_2_**), and 168 h (**e**,**f**), and graphic representation of the mass variation in PLGA/HA-MTX sphere coatings after testing under bio-simulated conditions (**g**).

Though coating alterations were locally identified after 48 h of testing, and local inter-particle connections were observed at 96 h and 168 h testing intervals only in nanosized spheres (<400 nm), no significant degradation of PLGA/HA-MTX spheres was noticed, as their microstructure was preserved up to one week after dynamic evaluation under bio-simulated conditions. As a general remark, an increase in the size and distribution of inorganic aggregates was noticed after increasing the immersion time (bright areas in SEM micrographs), an outcome of the gradual release of HA nanoparticles from PLGA spheres and the formation of new calcium phosphates. To support this latter premise, the collected EDS results (Figure 5) showed a time-depended increase in the amount of calcium phosphate aggregates (with mean Ca/P ratio of 1.42) and their more homogenous distribution onto the metallic substrate. This outcome indicated the presence of distinctive mineral deposits, most probably carbonated HA (confirming the more intense C–O stretching and bending vibrations), simultaneously resulting from the B-type substitution of HA by CO_3_^2−^ and the biomineralization of newly formed calcium carbonate. The formation of SBF-originating deposits was supported by the presence and uniform distribution of Mg and Na (after 24 h), but also Cl (after 48 h), onto the composite coatings. 

The biomineralization ability of PLGA/HA-MTX sphere coatings after one week of bio-simulated evaluation was also supported by the coatings’ mass variation (Figure 5g), which evidenced a gradual decrease in Δm (estimated as the mass difference between initial and bio-evaluated coatings) that was consequently transposed as a sustained increase in the mass of PLGA/HA-MTX sphere coatings. This outcome became more evident after prolonged testing times, as the post-immersion mass exceeds the pre-immersion mass of coatings starting from the second day of dynamic testing under biologically simulated conditions. 

### 3.2. In Vitro Biological Evaluation of Normal and Tumor Cell Interaction with Coatings

**Figure 6:**
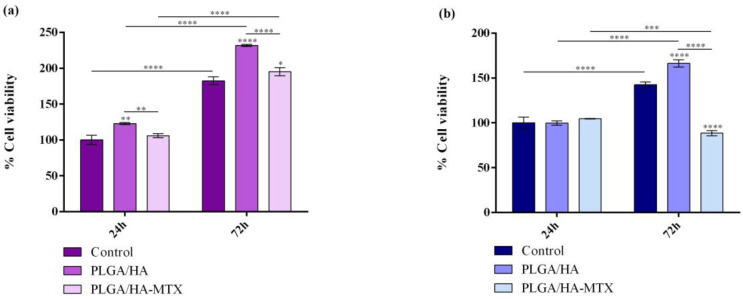
Graphical representation of cell viability and proliferation of (**a**) human preosteoblasts hFOB 1.19 and (**b**) human osteosarcoma Saos-2 cells after 24 h and 72 h of contact with PLGA/HA and PLGA/HA-MTX coatings as revealed by the MTT assay (* *p* value ≤ 0.05, ** *p* value ≤ 0.01, *** *p* value ≤ 0.001, **** *p* value ≤ 0.0001).

The impact of PLGA/HA-coated and PLGA/HA-MTX-coated samples on cell viability and proliferation potential of both normal and tumor cells was investigated by measuring the cell metabolic health through the MTT assay (Figure 6). The obtained results revealed notable variances in the viability and proliferative potential of normal and tumor cells after 24 h and 72 h of interaction with the novel coatings, specifically for MTX-loaded coatings. 

After 24 h, the cell viability of hFOB 1.19 cells was significantly increased on PLGA/HA-coated titanium compared to pristine (uncoated) titanium substrate. This trend toward increased cell viability also remained consistent after 72 h of culture. Moreover, the increase in cell viability between the two experimental time points revealed that hFOB 1.19 cells are capable of proliferating on both modified substrates, but they present a more pronounced proliferative activity when cultured on PLGA/HA coatings. For tumor cells, no significant changes in the cell viability of Saos-2 cells cultured on PLGA/HA coatings were observed after 24 h. After 72 h, findings similar to those of normal cells were noted for Saos-2 cells. These results highlight that the PLGA/HA coatings provide a more favorable environment for cells to interact with compared with pristine titanium, supporting better cellular attachment and growth in the case of both normal and tumor cells.

Regarding the cellular viability on PLGA/HA-MTX coatings, no significant differences were noted after 24 h in comparison with the cell viability of cells cultured on titanium for either cell type. Increasing the interaction period of human preosteoblasts with PLGA/HA-MTX coatings up to 72 h triggered a statistically significant increase in cell viability as compared with cell viability on uncoated titanium, accompanied by a decrease in the cell viability when compared with preosteoblasts cultured on PLGA/HA coatings. However, the proliferative capacity of human preosteoblasts was minimally affected, with the proportion of viable cells doubling at 72 h compared to 24 h of culture on PLGA/HA-MTX coatings. In contrast, tumor cells cultured for 72 h on PLGA/HA-MTX-coated samples exhibited a significantly decreased cellular viability compared to both uncoated samples and PLGA/HA coatings. Moreover, the PLGA/HA-MTX coatings severely impacted the tumor cells’ proliferative potential, with the cellular viability decreasing significantly after 72 h compared to 24 h.

**Figure 7:**
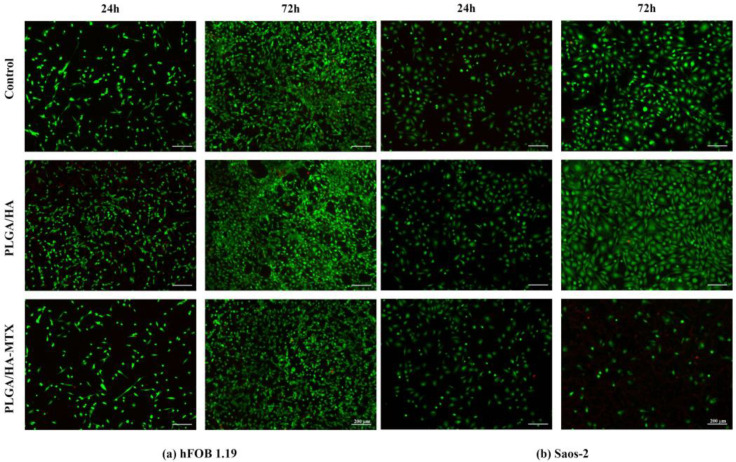
Fluorescence micrographs revealing live (green) and dead (red) (**a**) human preosteoblasts hFOB 1.19 and (**b**) human osteosarcoma Saos-2 cells after 24 h and 72 h of contact with the non-coated samples, PLGA/HA coatings, and PLGA/HA-MTX coatings (magnification 10×).

The Live/Dead assay (Figure 7) confirmed the findings obtained through the quantitative MTT assay. The visual examination of live and dead cells on the sample surfaces highlighted the ability of PLGA/HA coatings to enhance titanium surface properties, promoting cell adhesion and proliferation of both normal and tumor cells. Notably, no dead cells were identified on the PLGA/HA-coated surfaces at either time point, with a higher ratio of live cells on coated samples compared with uncoated titanium for both normal and tumor cells. Furthermore, after 24 h, a higher proportion of live cells presenting a flattened morphology was identified on the PLGA/HA coatings compared with pristine titanium, indicating an improved cellular adhesion of normal and tumor cells to PLGA/HA-coated substrates.

The addition of the chemotherapeutic drug within the coating’s structure had varying effects on the behavior of normal and tumor cells. Specifically, for human preosteoblasts, no dead cells were identified on the PLGA/HA-MTX coatings at any of the time points. After 24 h, the PLGA/HA-MTX coating’s live cell proportion was similar to that of the uncoated surfaces, but lower than in the case of PLGA/HA-coated surfaces. However, although after 72 h the ratio of live cells remained lower on PLGA/HA-MTX coatings compared to PLGA/HA coatings, no significant differences were noted compared to the control. Moreover, the fluorescence micrographs revealed the potential of PLGA/HA-MTX coatings to support hFOB 1.19 proliferation, with a significant increase in the live cells present on PLGA/HA-MTX coatings after 72 h compared to 24 h. Regarding the impact of PLGA/HA-MTX coatings on human osteosarcoma cells, no major differences were noticed after 24 h of culture. However, after 72 h of culture, dead cells were detected on PLGA/HA-MTX coatings, with a significant decrease in the living cells present on the material’s surface compared to the control and drug-free coatings, as well as compared with the proportion observed on PLGA/HA-MTX coatings after 24 h of culture.

**Figure 8:**
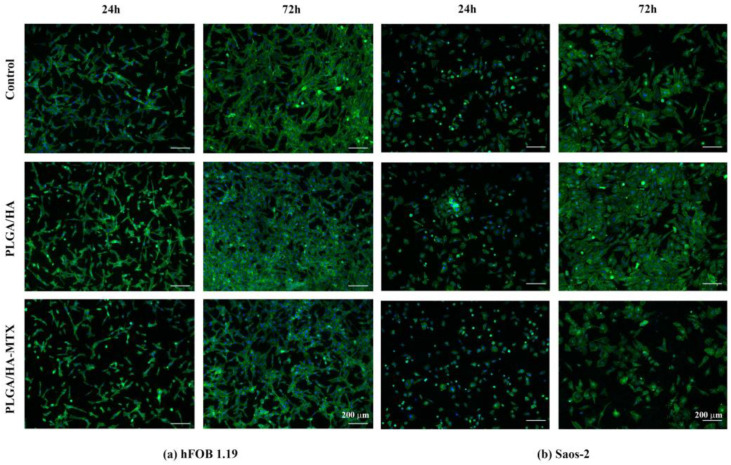
Fluorescence micrographs of the cell cytoskeleton of (**a**) human preosteoblasts hFOB 1.19 and (**b**) human osteosarcoma Saos-2 cells after 24 h and 72 h of contact with the non-coated samples, PLGA/HA coatings and PLGA/HA-MTX coatings, after staining of the actin filaments with phalloidin-FITC (green) and cell nuclei with DAPI (blue), (magnification 10×).

To assess the influence of PLGA/HA and PLGA/HA-MTX coatings on cell cytoskeleton structure and dynamics, F-actin filaments were stained with FITC-phalloidin and visualized *via* fluorescence microscopy (Figure 8). After 24 h, hFOB 1.19 cells exhibited their typical polygonal morphology on the PLGA/HA-coated surfaces, with long well-organized F-actin filaments surrounding the cellular nuclei. Furthermore, the cells displayed a flattened appearance, indicating strong substrate adhesion. Similarly, most preosteoblasts cultured on PLGA/HA-MTX coatings exhibited these features, although a minority of cells appeared round, suggesting delayed adhesion to the coatings in the presence of methotrexate. However, after 72 h of culture, no differences were observed between coated samples, as hFOB 1.19 cells displayed a well-developed cytoskeleton with defined F-actin filaments contributing to cell–cell interactions and the formation of 3D cellular networks. 

Significant differences were observed among osteosarcoma cell samples, even after 24 h of culture. While Saos-2 cells cultured on PLGA/HA-coated surfaces exhibited a spindle-like shape and a well-developed cytoskeleton, the cells cultured on PLGA/HA-MTX coatings failed to adopt their typical morphology and exhibited weak adhesion to the substrate, appearing rounded with a less developed cytoskeleton. After 72 h, the Saos-2 cells adopted their characteristic morphology on all sample surfaces, though the addition of MTX induced significant changes in F-actin filament distribution, with fibers mainly concentrated at the cell periphery, resulting in an overall less developed cytoskeleton. 

The biological investigations revealed that the novel PLGA/HA coatings have excellent biocompatibility and promote enhanced cell adhesion and proliferation when compared with pristine titanium. These findings were attributed to the constituent components employed for coating fabrication, both widely used for bone tissue engineering strategies due to their biocompatibility, biodegradability, and osteogenic ability [92,93,94], and lined up to previous studies regarding the excellent biocompatibility of such composites. While preserving their drug-releasing ability to promote and support local pharmacological effects, nano-HA-modified PLGA particles exhibited osteoconductive and osteoinductive effects with respect to mesenchymal stem cells and preosteoblasts, being validated as osteogenic candidates for bone repair and regeneration [81,88,89]. Similar cellular effects have been reported for nano-HA/PLGA composite coatings proposed for improving the osseointegration of metallic implants while modulating local inflammation and infection [84,95]. More than exerting drug-eluting abilities and promoting osteogenic events, nanostructured PLGA/HA scaffolds demonstrated suitable mechanical and microstructural compliance for bone repair and new bone formation [82,86]. Furthermore, fabricating three-dimensional (3D) constructs for the repair and replacement of bone tissue provides indisputable advantages in terms of patient compliance, composition-related multifunctionality, process scalability, and cost efficiency [96,97]. Besides enabling the accurate customization of the product, additive manufacturing facilitates the high-yield fabrication of implantable devices with complex geometries and compositions that precisely and selectively fulfill the physiological and therapeutic requirements [98,99]. In this regard, nanostructured PLGA/HA 3D scaffolds have been evaluated as suitable architectures for the attachment, migration, normal development, and osteogenic differentiation of mesenchymal stem cells while exerting superior new bone formation and angiogenic ability in animal models [87,100,101]. Herein, surfaces modified with PLGA/HA sphere coatings proved superior substrates for cellular interactions with both normal and tumor cells compared to pristine medical-grade titanium, starting from the initial evaluation point of 24 h and up to 72 h, as evidenced in terms of viability and proliferation (through the metabolic health) and in terms of adhesion, proliferation, and development (through the microscopy studies).

Moreover, the PLGA/HA-MTX coatings proposed here determined no significant cellular events in preosteoblasts after 24 h, with increased proliferation and enhanced development after 72 h when compared to pristine titanium and slightly altered cellular events compared to PLGA/HA-coated samples. The short-time incubation of osteosarcoma cells in the presence of PLGA/HA-MTX-coated titanium caused no major differences in terms of cellular viability and proliferation but resulted in weak adhesion and slightly affected cytoskeleton development when compared to the control. By contrast, their prolonged interaction determined important cellular alterations, as revealed by the decreased cellular viability and proliferative potential, increased cellular death, and significantly affected cellular development. Considering the drug release prolife from PLGA/HA-MTX sphere coatings, we could conclude that the released MTX (at 9.5 μg/mL concentration) was not enough to prominently alter the Saos-2 cells after one day, while the amount of ~18 μg/mL induced important cytotoxicity within the osteosarcoma cells after 3 days. Our findings are compliant with the results reported for HA-doxorubicin-chitosan/polycaprolactone-MTX nanoparticles. Under physiological pH evaluation, a cumulative MTX release of ~9.9 μg/mL (22%) and ~11 μg/mL (24%) was recorded after 24 h and 72 h, respectively, which determined a time-dependent and dose-dependent cytotoxicity in MG-63 osteosarcoma cells [102]. Using the same human tumor cell line, more pronounced cytotoxic effects were evidenced for PLGA nanocarriers in lower doses when compared to pure MTX in higher doses. Also, a time-dependent MTX uptake was noticed in MG-63 cells, with intracellular concentrations of 14.15 μg/mL and 11.24 μg/mL after 24 h, corresponding to PLGA-MTX and PLGA-nanoceramic-MTX formulations, respectively [103]. After 72 h, a drastic decrease in the viability of Saos-2 cells of ~40% and ~50% was observed for PLGA/HA-MTX-coated surfaces when compared to pristine and PLGA/HA-coated titanium, respectively, highlighting the selective toxicity of MTX-loaded sphere coatings against tumor cells. Likewise, significant cellular death was reported after 3 days in MG-63 cells treated with MTX-loaded and MTX-conjugated PVA-HA nanoparticles, the cytotoxic effects being more prominent for the formulations in which the drug was physically loaded [56]. While they exhibited reduced toxic effects in normal cells, MTX-conjugated PEG/nano-HA systems determined lower effective concentrations than pure MTX in three osteosarcoma cell lines [57].

Our results confirmed the therapeutic potential of sphere coatings to regulate the beneficial outcomes of implantable surfaces for bone cancer applications. While promoting and supporting normal cellular events in healthy preosteoblasts, our studies revealed the selective cytotoxicity of methotrexate-loaded composite coatings towards osteosarcoma cells. This is mainly due to exerting drug-releasing ability and time-dependent anti-cancer effects. For a thorough evaluation of the complex cellular events of the PLGA/HA-MTX sphere coatings, further studies on the pro-osteogenic activity will be considered. Furthermore, long-term bone-repair and bone-remodeling effects could also be evaluated in a prospective animal model validation of the novel materials. The facile, cost-effective, and scalable protocol proposed here allows for the surface modification of devices with different compositions and geometries, including bone fixation devices and bone implants. Therefore, the PLGA/HA-MTX sphere coatings represent viable candidates for the management of cancerous patients that undergo bone surgery by modulating the therapeutic outcomes of inserted devices.

## 4. Conclusions

Incorporating MTX in the coating’s structure repurposes the biomaterial from bone regeneration applications to an effective strategy for osteosarcoma management. Our results revealed that the novel PLGA/HA-MTX sphere coatings exhibit selective cytotoxicity towards osteosarcoma tumor cells while exerting minimal impact on normal preosteoblasts. Therefore, the novel drug-loaded coating represents a potential bone substitute material for the delivery of therapeutic molecules, which could minimize the adverse effects associated with the free-MTX treatment.

## Data Availability

Data are contained within the article.
